# Difference in Restricted Mean Survival Time for Cost-Effectiveness Analysis Using Individual Patient Data Meta-Analysis: Evidence from a Case Study

**DOI:** 10.1371/journal.pone.0150032

**Published:** 2016-03-09

**Authors:** Béranger Lueza, Audrey Mauguen, Jean-Pierre Pignon, Oliver Rivero-Arias, Julia Bonastre, MAR-LC Collaborative Group

**Affiliations:** 1 Gustave Roussy, Service de biostatistique et d’épidémiologie, Villejuif, France; 2 CESP, INSERM U1018, Université Paris-Sud, Université Paris-Saclay, Villejuif, France; 3 Gustave Roussy, Ligue Nationale Contre le Cancer meta-analysis plateform, Villejuif, France; 4 University of Oxford, National Perinatal Epidemiology Unit, Nuffield Department of Population Health, Oxford, United Kingdom; 5 Red de Investigación en Servicios de Salud en Enfermedades Crónicas (REDISSEC), Madrid, Spain; Cardiff University, UNITED KINGDOM

## Abstract

**Objective:**

In economic evaluation, a commonly used outcome measure for the treatment effect is the between-arm difference in restricted mean survival time (rmstD). This study illustrates how different survival analysis methods can be used to estimate the rmstD for economic evaluation using individual patient data (IPD) meta-analysis. Our aim was to study if/how the choice of a method impacts on cost-effectiveness results.

**Methods:**

We used IPD from the Meta-Analysis of Radiotherapy in Lung Cancer concerning 2,000 patients with locally advanced non-small cell lung cancer, included in ten trials. We considered methods either used in the field of meta-analysis or in economic evaluation but never applied to assess the rmstD for economic evaluation using IPD meta-analysis. Methods were classified into two approaches. With the first approach, the rmstD is estimated directly as the area between the two pooled survival curves. With the second approach, the rmstD is based on the aggregation of the rmstDs estimated in each trial.

**Results:**

The average incremental cost-effectiveness ratio (ICER) and acceptability curves were sensitive to the method used to estimate the rmstD. The estimated rmstDs ranged from 1.7 month to 2.5 months, and mean ICERs ranged from € 24,299 to € 34,934 per life-year gained depending on the chosen method. At a ceiling ratio of € 25,000 per life year-gained, the probability of the experimental treatment being cost-effective ranged from 31% to 68%.

**Conclusions:**

This case study suggests that the method chosen to estimate the rmstD from IPD meta-analysis is likely to influence the results of cost-effectiveness analyses.

## Introduction

The individual patient data meta-analysis (IPD-MA) has become the gold standard for obtaining the best evidence for treatment effects (e.g. see [1,2]). The aim of an IPD-MA is to estimate a pooled treatment effect from the aggregation of data from all randomised controlled trials (RCTs) that investigated the same clinical question [3–5]. This pooled treatment effect is a relative outcome measure often expressed for survival data as a pooled hazard ratio. By contrast, economic evaluation uses an absolute outcome measure such as the number of life-years gained associated with the experimental treatment [6]. It is estimated as the between-arm difference in the restricted mean survival time (rmstD) and corresponds to the area between the two survival curves for the experimental arm and the control arm restricted to a certain time horizon [7].

Economic evaluations based on IPD-MA raise methodological concerns because of data clustering (patients within trials) which must be considered in the analysis. Some economic studies have already used IPD-MA [8–10]. However, these studies failed to acknowledge data clustering or did not justify the choice of the method used to estimate the rmstD. As a matter of fact, research on methods used to conduct economic evaluation based on IPD-MA is still in its infancy [11–14]. Issues raised by the estimation of the rmstD for economic evaluation from a trial have already been investigated but none of these studies dealt with the use of IPD-MA [15–17]. Conversely, in a recent paper, Wei and colleagues [18] compared three different methods to estimate the rmstD from IPD-MA but they did not study cost-effectiveness.

In this case-study, we illustrate how different survival analysis methods can be used to estimate the rmstD for economic evaluation using IPD-MA. Our aim is to study if/how the choice of a survival analysis method impacts on the cost-effectiveness results.

## Data

We had access to the patient-level data from the Meta-Analysis of Radiotherapy in Lung Cancer (MAR-LC) collaborative group [19] which was previously used in a Dutch economic evaluation [20]. The MAR-LC comprised 2,000 distinct patients with a non-metastatic non-small cell lung cancer treated with radiotherapy and who had been enrolled in ten distinct phase III RCTs [19]. MAR-LC trials compared conventional radiotherapy (RT) regimen with modified RT regimen and are listed in S1 Table. Of note, two trials were each split into two separate comparisons which correspond to strata of these trials with patients receiving or not chemotherapy (PMCI 88C091 CT and PMCI 88C091; CHARTWEL CT and CHARTWEL). Modified RT included hyperfractionated RT which consists in increasing the number of fractions per day with a decreased dose per fraction, and/or accelerated RT, in which the overall treatment time is reduced. The MAR-LC primary endpoint was overall survival. In our analysis, the time horizon was restricted to 5 years to be consistent with the follow-up of the MAR-LC trials and also as it was a time point of clinical interest in MAR-LC [19].

## Methods

### Estimation of the difference in restricted mean survival time from IPD meta-analysis

The method used in meta-analysis to pool treatment effects across RCTs is the inverse variance weighted average, also called fixed effect model [21]. It is a two-stage method which is based on the estimation of the treatment effect, firstly, in each RCT and secondly, the aggregation of estimates [22–24]. The purpose is to give more weight to trials that yield more information about the treatment effect and thus have a lower variance. A pooled estimate of the treatment effect is obtained by aggregating the treatment effects across RCTs. This aggregation method ensures the correct comparisons of patients within each RCT (stratification by trial) and therefore an unbiased estimation of the pooled treatment effect. It can also account for a potential difference in the treatment effect between trials (between-trial heterogeneity). The treatment effects to be pooled are mostly expressed in terms of log odds ratios or log hazard ratios for survival endpoints [21]. Conversely, a common outcome measure in economic evaluation is the difference in the restricted mean survival time (rmstD) [6,7]. This absolute outcome can be expressed as the number of life-years gained associated with the treatment.

### Selection of survival analysis methods

In order to estimate the rmstD from IPD-MA, we considered methods used by Wei and colleagues [18] and chose to adapt other non-parametric methods that are applied in the field of IPD-MA. All these methods have never been applied to assess the rmstD for economic evaluation. Methods were classified into two approaches. With the first approach, the rmstD is estimated directly as the area between the two pooled survival curves. With the second approach, the rmstD is based on the aggregation of rmstDs estimated in each RCT [18]. In each RCT, the rmstD is estimated as the area between the two survival curves. The rmstDs are then pooled across trials. This second approach is an extension of the inverse variance weighted average method that is classically used in meta-analysis to pool treatment effects across RCTs.

#### Approach 1: Area between the two pooled survival curves

So far, two non-parametric methods (Stewart-Parmar and Peto methods) have been used for estimating pooled survival curves from IPD meta-analysis [25,26]. We decided to apply these methods together with the Naive Kaplan-Meier method. The Naive Kaplan-Meier method considers the IPD from the different RCTs as if they originated from a unique RCT. The rmstD is then the area between the two Kaplan-Meier survival curves. This method does not assume proportional hazards, but neither stratification by trial nor heterogeneity of treatment effect can be taken into account to estimate the pooled survival curves. Stewart-Parmar and Peto methods are based on the aggregation of the hazard ratios across RCTs using the inverse variance weighted average. We applied Stewart and Parmar methodology [25] to estimate the pooled survival curve for the experimental arm using the pooled hazard ratio and the naive Kaplan-Meier survival curve in the control group. With this method, stratification by trial and treatment effect heterogeneity are addressed but the treatment effect is assumed to be constant over time (proportional hazards assumption). Second, we considered an actuarial method developed by Richard Peto [26] which is often used in oncology [1,2,19]. Survival probabilities in each arm are estimated at predetermined time intervals from the pooled hazard ratio, which may vary between time periods, and from the survival probability of the whole population (both control and experimental arms). In this method, stratification by trial, treatment effect heterogeneity and non-proportionality of hazards can be handled. We computed this method using three different time interval definitions: one year, one month and an interval length based on the quintiles of the distribution of deaths in the whole population.

#### Approach 2: Pooling differences in restricted mean survival time

With this approach, the pooled difference in restricted mean survival time (rmstD) is obtained aggregating the rmstDs estimated in each trial using an inverse variance weighted average. In each trial, the rmstD can be estimated using different survival analysis methods. We decided to consider the Kaplan-Meier method and parametric survival analysis models. The selection of the parametric model was based upon the log-likelihood ratio test and log-cumulative hazard plots [17]. We retained the exponential model. The Pooled Kaplan-Meier and Pooled Exponential methods deal with stratification by trial and treatment effect heterogeneity. The Pooled Kaplan-Meier method addresses non-proportional hazards, whereas the Pooled Exponential method, which is based on the exponential proportional hazards model, does not.

Details on the methods are provided in Table 1 and in the S1 Supporting Information. Table 1 summarizes the ability of these methods to address stratification by trial, non-proportionality of hazards (variation of the treatment effect over time) and treatment effect heterogeneity.

**Table 1:** Characteristics of the survival analysis methods used to estimate the rmstD from IPD meta-analysis.

Survival methods		Accounts for			Difference in restricted mean survival time
		Stratification on trial	Potential treatment effect heterogeneity	Non-proportional hazards	
**Approach 1**	**Naive Kaplan-Meier**	No	No	Yes	
	**Stewart-Parmar**	Yes	Yes^$^	No	
	**Peto-year**	Yes	Yes^$^	Yes^£^	$rmstD=\int_{0}^{t^{*}}{\text{S}}_{\text{Exp},\;\text{pooled}}\;(\text{t}).\text{dt}-\int_{0}^{t^{*}}{\text{S}}_{\text{Control},\;\text{pooled}}\;(\text{t}).\text{dt}$
	**Peto-month**	Yes	Yes^$^	Yes^£^	
	**Peto-quintiles**	Yes	Yes^$^	Yes^£^	
**Approach 2**	**Pooled Kaplan-Meier**	Yes	Yes^$^	Yes	Inverse variance weighted average to pool rmstD_j_ estimated in each trial *j*
	**Pooled Exponential**	Yes	Yes^$^	No	$rmstD_{j}=\int_{0}^{t^{*}}{\text{S}}_{\text{Exp},\;\text{j}}(\text{t}).\text{dt}-\int_{0}^{t^{*}}{\text{S}}_{\text{Control},\;\text{j}}(\text{t}).\text{dt}$

HR_pooled_: pooled Hazard Ratio; MA: Meta-Analysis; rmstD: difference in restricted mean survival time; *S*_*Exp*,*pooled*_: pooled survival function for the experimental arm; *S*_*Control*,*pooled*_: pooled survival function for the control arm; *S*_*Exp*,*j*_: survival function for the experimental arm in trial *j*; *S*_*Control*,*j*_: survival function for the control arm in trial *j*

^$^: treatment effect heterogeneity can be addressed through the two-stage model used to pool the trial-specific HRs (Stewart-Parmar and Peto methods) or rmstDs (Pooled Kaplan-Meier and Pooled Exponential)

^£^: *HR*_*pooled*_ can vary between time periods

Except for the Naïve Kaplan-Meier method, all survival analysis methods were not available in standard statistical softwares. We coded the methods using R version 3.1.3 (R Foundation, Vienna, Austria) and SAS version 9.3 (SAS Institute, Cary, NC). Code is available from the authors upon request.

### Cost-effectiveness analysis

Direct costs (radiotherapy (RT), medical transportation, disease progression and esophagitis) were assessed at the patient level using the healthcare resource use measured in the MAR-LC. The costs were estimated in the French context from a payer’s perspective and expressed in 2012 euros. In each trial, the mean cost per patient for RT and medical transportation were estimated from the number of RT fractions received. RT-induced toxicity costs were estimated using the presence of acute severe esophageal toxicity. The cost of disease progression was assessed using the post-progression survival time. The unit costs were extracted from the literature for medical transportation [27] and disease progression costs [28]. Radiotherapy and acute esophagitis unit costs were computed as the mean lump sum per corresponding diagnosis-related group in the French prospective payment scheme.

The results of the cost-effectiveness analysis were presented using the incremental cost-effectiveness ratios (ICER) expressed as the cost per life-year gained and cost-effectiveness acceptability curves [29]. The ICER was defined as the difference in mean costs between the two types of radiotherapy regimen (modified and conventional) divided by the rmstD. Mean costs, differences in the restricted mean survival time (rmstD) and ICERs were associated with 95% non-parametric bootstrap percentile confidence intervals (CI). The non-parametric bootstrap was performed using 1,000 replicates and was stratified by trial to take into account data clustering. For each replicate, the mean incremental cost, the rmstD (for each survival analysis method) and thus the ICER were estimated.

## Results

MAR-LC included 1,849 deaths, 1,777 (96%) of which occurred during the first five years, corresponding to a survival probability at five years of 9% [19]. Modified RT was associated with longer overall survival (pooled hazard ratio = 0.88, 95% CI: [0.80–0.97], p = 0.009). The overall proportional hazard assumption was verified in the meta-analysis (p = 0.91) as well as in individual trials according to Wei and colleagues’ approach [18]. There was no treatment effect heterogeneity between trials (p = 0.37, Higgins I² = 8%).

The mean total cost per patient was € 25,331 (95% CI: € 23,630–€ 27,115) for conventional RT and € 29,659 (95% CI: € 27,845–€ 31,507) for modified RT, corresponding to a mean incremental cost of € 4,328 (95% CI: € 1,830–€ 6,804).

**Fig 1:**
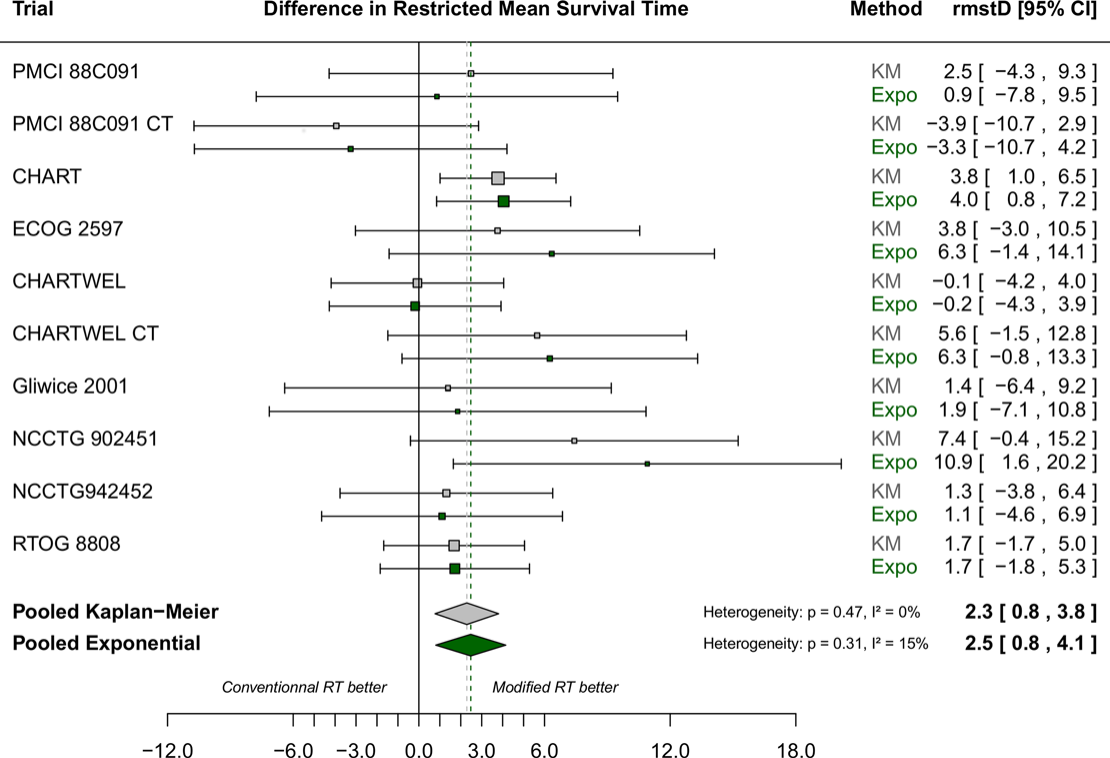
Forest plot for differences in restricted mean survival time estimated using Pooled Kaplan-Meier (grey squares and diamond) and Pooled Exponential (dark green squares and diamond) applied to the MAR-LC dataset. Each trial is represented by a square, the center of which denotes the difference in restricted mean survival time (rmstD) for that trial comparison, with the horizontal lines showing the 95% CIs. The size of the square is directly proportional to the amount of information contributed by the trial. The diamonds represent overall rmstDs, with the center denoting the rmstD and the extremities the 95% CI. CHART: Continuous Hyperfractionated Accelerated Radiation Therapy; CHARTWEL: CHART Week-End Less; CI: confidence interval; CT: chemotherapy; ECOG: Eastern Cooperative Oncology Group; Expo: Exponential; KM: Kaplan-Meier; MAR-LC: Meta-Analysis of Radiotherapy in Lung Cancer; NCCTG: North Central Cancer Treatment Group; PCMI: Peter MacCallum Institute; rmstD: difference in restricted mean survival time; RTOG: Radiation Therapy Oncology Group; RT: Radiotherapy.

Survival curves for the two arms in MAR-LC estimated using Naive Kaplan-Meier and Stewart-Parmar, Peto-month, Peto-year and Peto-quintiles are respectively shown in S1–S4 Figs. Naive Kaplan-Meier and Stewart-Parmar provided the same survival curve, by definition, for the conventional arm, and quite similar survival curves for the modified arm (S1 Fig). Peto-month, Peto-year and Peto-quintiles survival curves differed as they were not based on the same time interval (S2–S4 Figs). Fig 1 shows the forest-plot for the difference in restricted mean survival time (rmstD) estimated using Kaplan-Meier or the exponential model for each of the ten RCTs in MAR-LC, demonstrating no heterogeneity between trials (p = 0.47, Higgins I² = 0% for Pooled Kaplan-Meier and p = 0.31, Higgins I² = 15% for Pooled Exponential). The rmstDs estimated using the different survival analysis methods are shown in Table 2. They ranged from 1.7 month in the Peto-quintiles method to 2.5 months in the Pooled Exponential method. As the month intervals contained fewer events, the variance of the rmstD was higher in the Peto-month method compared to the Peto-year and Peto-quintiles methods. Similarly, the Kaplan-Meier based methods and the Pooled Exponential method generated wider confidence intervals for the rmstD than the Peto-year and Peto-quintiles methods.

**Table 2:** Difference in restricted mean survival time and ICER according to the survival analysis method.

Survival methods		Difference in restricted mean survival time (in month) [95% CI]	Mean ICER (cost per life year gained) [95% CI]
**Approach 1**	**Peto-quintiles**	1.7 [0.4–3.1]	€ 34,934 [12,506–98,066]
	**Peto-year**	1.8 [0.5–3.0]	€ 33,387 [13,512–75,753]
	**Stewart-Parmar**	2.1 [0.6–3.5]	€ 29,017 [11,822–68,206]
	**Naive Kaplan-Meier**	2.2 [0.6–3.7]	€ 26,848 [11,152–68,297]
	**Peto-month**	2.3 [0.7–3.9]	€ 28,022 [10,563–61,608]
**Approach 2**	**Pooled Kaplan-Meier**	2.3 [0.7–3.8]	€ 25,527 [10,355–66,289]
	**Pooled Exponential**	2.5 [0.7–4.2]	€ 24,299 [9,584–59,119]

CI: confidence interval; ICER: Incremental Cost-Effectiveness Ratio; rmstD: difference in restricted mean survival time

**Fig 2:**
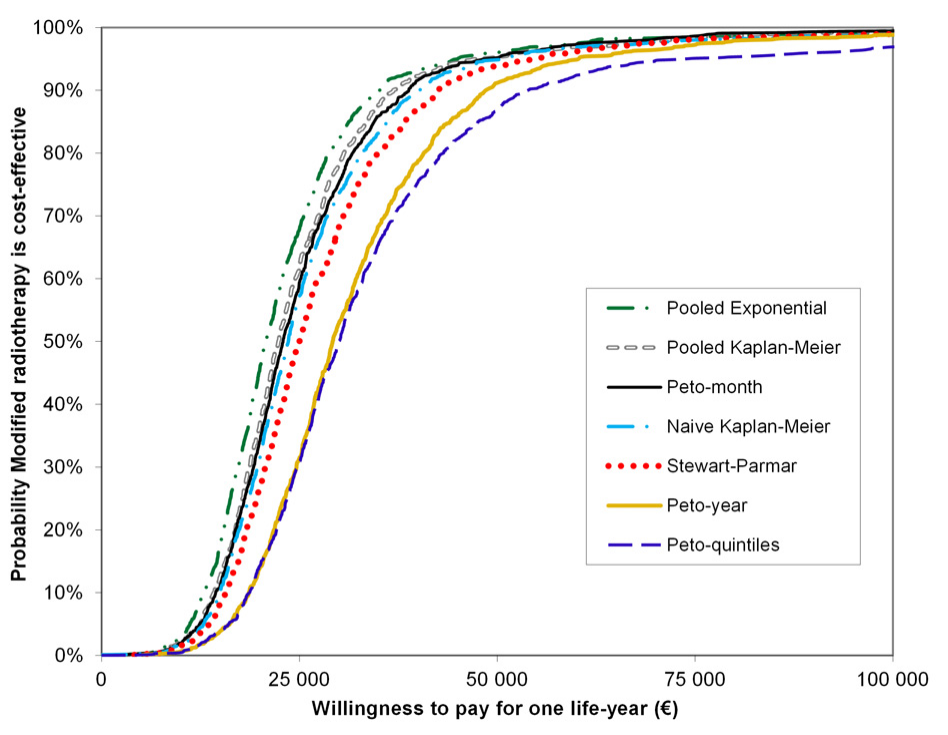
Acceptability curves showing the probability that modified radiotherapy is cost-effective at different thresholds of willingness-to-pay for one life year. Cost-effectiveness acceptability curves were derived from the 1,000 ICERs based on the bootstrap replicates to illustrate the uncertainty surrounding the cost-effectiveness of the experimental arm radiotherapy. Modified RT is considered cost-effective if the ICER is less than the willingness-to-pay for one life year. The acceptability curve represents the proportion of the replicates where modified RT is cost-effective for a range of different willingness-to-pay.

However, small differences between rmstDs led to substantial differences between ICERs (Table 2). Mean ICERs ranged from € 24,299 to € 34,934 per life-year gained, respectively for the Pooled Exponential and the Peto-quintiles methods (Table 2). In each bootstrap replicate, modified RT was both more effective—irrespective of the survival analysis method used—and more expensive than conventional RT. The acceptability curve of the Pooled Exponential method was above the six other methods (Fig 2). Pooled Kaplan-Meier, Peto-month, naive Kaplan-Meier and Stewart-Parmar acceptability curves were similar whereas the acceptability curves based on the Peto-year and Peto-quintiles methods were notably lower than the others. Using a willingness to pay for one life-year gained above € 50,000, all the methods concluded that modified RT was cost-effective with a probability of approximately 90%, whereas below € 50,000, acceptability curves could lead to different conclusions. With a ceiling ratio of € 25,000 per life-year gained, the probability of modified RT being cost-effective ranged from 31% with Peto-quintiles to 68% with the Pooled Exponential method (Fig 2).

## Discussion

Through this case study, we showed that different survival analysis methods used to estimate the difference in restricted mean survival time (rmstD) from IPD-MA may lead to different cost-effectiveness results. This may be explained by two factors. First, we showed that the survival analysis methods have different abilities to address the specificities of the hierarchical structure of IPD meta-analysis. The Peto and Pooled Kaplan-Meier methods are the only methods that account for stratification by trial, treatment effect heterogeneity and non-proportionality of hazards. The Pooled Kaplan-Meier method and the Pooled Exponential method enable us to study the heterogeneity of the rmstDs across trials (Fig 1). Second, time interval definition used in each method also influences cost-effectiveness results. Survival probabilities are estimated after each event in the naive Kaplan-Meier, Pooled Kaplan-Meier, and Stewart-Parmar methods. In the Peto-month method, survival probabilities are estimated every month which is quite similar to estimations at each event. Similarly, in the Pooled Exponential method, all observations (at any time) are used to fit the best model. These methods lead to the most optimistic acceptability curves. By contrast, the Peto-year and the Peto-quintiles methods yield different results because they are based on larger time intervals which provide less uncertainty in the rmstD estimation, possibly at the cost of being biased as they provide notably lower estimations for the rmstD as compared to the other studied methods.

One previous study highlighted the importance of the choice of a survival analysis model both in cost-effectiveness analyses (CEAs) alongside RCTs and for CEAs based on meta-analysis [30]. Guyot and colleagues [30] pointed out that survival outcome in CEAs should be estimated with the same statistical method used for efficacy. That is why our focus was mostly on non-parametric methods used to estimate efficacy in the field of IPD-MA and why we dismissed other parametric methods proposed in the literature to estimate pooled survival curves [31]. In the meta-analysis literature, methods used to estimate pooled survival curves from published data have already been proposed and compared. Earle and Wells [32] compared five methods to combine published survival curves from studies of patients treated with chemotherapy for advanced non-small-cell lung cancer. The authors concluded that, overall, all the five methods were quite accurate but they pointed out that most of these methods failed to address stratification by trial and treatment effect heterogeneity. These methods were developed for summary data and are not applicable to IPD meta-analysis. In a recent paper, Wei and colleagues used the same two-stage approach as our second approach in which the rmstDs are estimated in each trial and are then aggregated [18]. They compared three methods of estimation of the trial-specific rmstD: the “Integrated difference of survival functions” method, which is equivalent to the Pooled Kaplan-Meier method, a pseudo values method and a flexible parametric survival model. Based on two applications and a simulation study, the authors concluded that the three methods yielded similar results with respect to bias, mean square error and coverage probability. This is consistent with our findings in which the Pooled Kaplan-Meier and Pooled Exponential methods led to similar rmstD estimations (Fig 1). We did not consider the non-parametric pseudo-values method, but Wei *et al*. [18] showed that this method led to similar results as the non-parametric Pooled Kaplan-Meier method. With the second approach, we selected one parametric model as Wei *et al* [18]. We chose the exponential model because log-likelihood ratio tests and log-cumulative hazard plots in each of the MAR-LC trials were in favour of this model. Furthermore, the issue of comparing different parametric models was beyond the scope of this paper, and has already been explored in the literature [15–17].

Commonly to other case studies, our results were driven by the characteristics of our clinical data. Even though, there was no treatment effect heterogeneity between MAR-LC trials and survival hazards were proportional, we noted a difference in mean ICERs generated by the methods. The difference may be even larger in case of treatment effect heterogeneity or non-proportionality of hazards.

The estimation of the overall rmstD depends on the choice of the time horizon t* which is still debated in the literature [7,18]. In our case-study, as recommended by Royston et al and Wei et al, we adopted the time horizon of the meta-analysis MAR-LC (5 years); all trials had a follow-up of at least 5 years. However, the trials included in a meta-analysis may have different lengths of follow-up. There is thus a compromise to achieve between a too short time horizon that would not take into account all information from all trials, and a too long time horizon that would necessitate the use of parametric extrapolation (see below) for most of the trials in the meta-analysis. When different lengths of follow-up is an issue (e.g. in case of non-proportional hazards with survival curves crossing later than t*), a sensitivity analysis varying t* should be performed to determine the impact on the estimation of the overall rmstD.

There is currently a debate about when and how to extrapolate survival curves up to a lifetime horizon for economic evaluations [15–17]. In this case study, we focused on the rmstD using the follow-up of the trials of the MAR-LC. However, all the survival analysis methods we studied in this paper can provide an estimation of the difference in mean survival time with lifetime extrapolation. For the first approach, the rmstD can be estimated based on the follow-up of the trials using pooled survival curves. Then, parametric models can be used to estimate the difference in mean survival time beyond the trials’ follow-up. Alternatively, similarly to the Stewart-Parmar method and to the method used in a number of studies reviewed by Guyot and colleagues [30], one could fit a parametric model to compute the survival function in the control arm. Then, one could use a pooled hazard ratio to derive the survival function in the experimental arm. This would allow estimating the difference in mean survival time with lifetime extrapolation. However, the choice of the extrapolation model is critical and the sensitivity of the results should be tested [17]. For the second approach, with the Pooled Kaplan-Meier method, difference in mean survival time could be estimated for each trial using Kaplan-Meier curves with extrapolated parametric [33] or non-parametric [34] tails.

## Conclusion

This case study showed that the choice of survival analysis method to estimate the difference in restricted mean survival time from an IPD meta-analysis is likely to exert an impact on cost-effectiveness results. It appears that the Pooled Kaplan Meier method exhibits many advantages. First, it addresses stratification by trial, treatment effect heterogeneity, and non-proportionality of hazards. Second, unlike the actuarial Peto method, it does not rely on any time interval definition. Finally, this method allows studying the potential heterogeneity of rmstD across trials and has been proved to be unbiased and with a good coverage probability (Wei et al, 2015). Our future prospects include a simulation study in order to be able to generalize the results found in this case study.

## Supporting Information

S1 Fig Survival curves estimated using the Naive Kaplan-Meier method and the Stewart-Parmar method applied to the MAR-LC dataset. 944 patients in the conventional radiotherapy arm and 1,046 in the modified radiotherapy arm. MAR-LC: Meta-Analysis of Radiotherapy in Lung Cancer; RT: Radiotherapy.
(TIF)

S2 Fig Survival curves estimated using the Peto-month method applied to the MAR-LC dataset. (TIF)

S3 Fig Survival curves estimated using the Peto-year method applied to the MAR-LC dataset. (TIF)

S4 Fig Survival curves estimated using the Peto-quintiles method applied to the MAR-LC dataset. Estimations were done every 355 deaths: at 0.45 year, 0.81 year, 1.25 year, 2.02 years, 5 years and an extra point estimation for patients who died after 5 years.
(TIF)

S1 Supporting Information Methodological details. (PDF)

S1 Table List of trials included in the Meta-Analysis of Radiotherapy in Lung Cancer. CHART: Continuous Hyperfractionated Accelerated Radiation Therapy; CHARTWEL: CHART Week-End Less; CI: confidence interval; CT: chemotherapy; ECOG: Eastern Cooperative Oncology Group; HR: Hazard ratio for Modified RT versus Conventional RT; MAR-LC: Meta-Analysis of Radiotherapy in Lung Cancer; NCCTG: North Central Cancer Treatment Group; PCMI: Peter MacCallum Institute; RTOG: Radiation Therapy Oncology Group; RT: Radiotherapy; *: see reference [19] for further details and for the trials references.
(PDF)
