# Supplementary figures and images for: Difference in Restricted Mean Survival Time for Cost-Effectiveness Analysis Using Individual Patient Data Meta-Analysis: Evidence from a Case Study
Source: PLoS One. 2016 Mar 9;11(3):e0150032. doi: 10.1371/journal.pone.0150032 (PMC4784740; doi:10.1371/journal.pone.0150032)

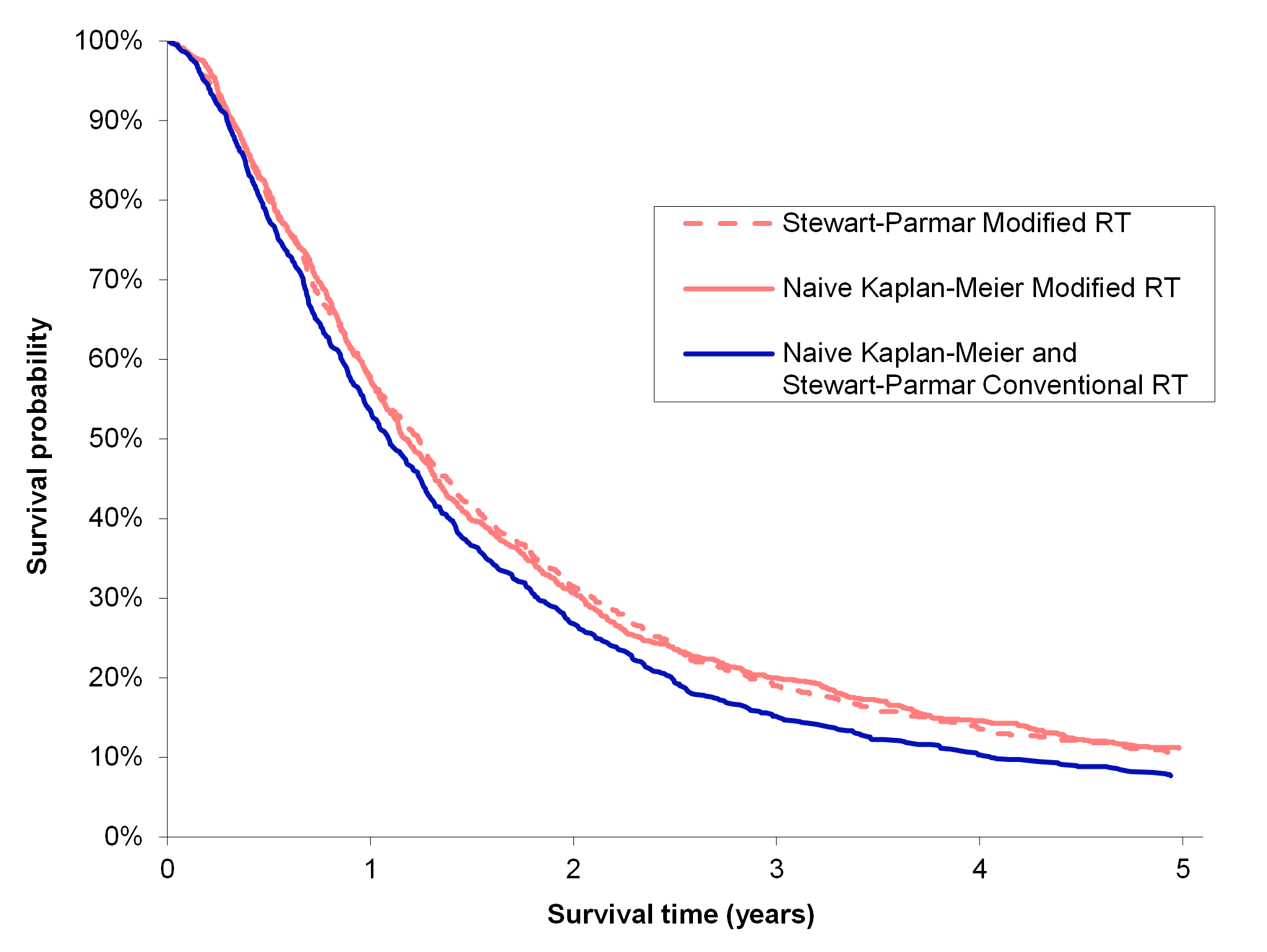

Supplement: S1 Fig — 944 patients in the conventional radiotherapy arm and 1,046 in the modified radiotherapy arm. MAR-LC: Meta-Analysis of Radiotherapy in Lung Cancer; RT: Radiotherapy. (TIF) [file pone.0150032.s001.tif]

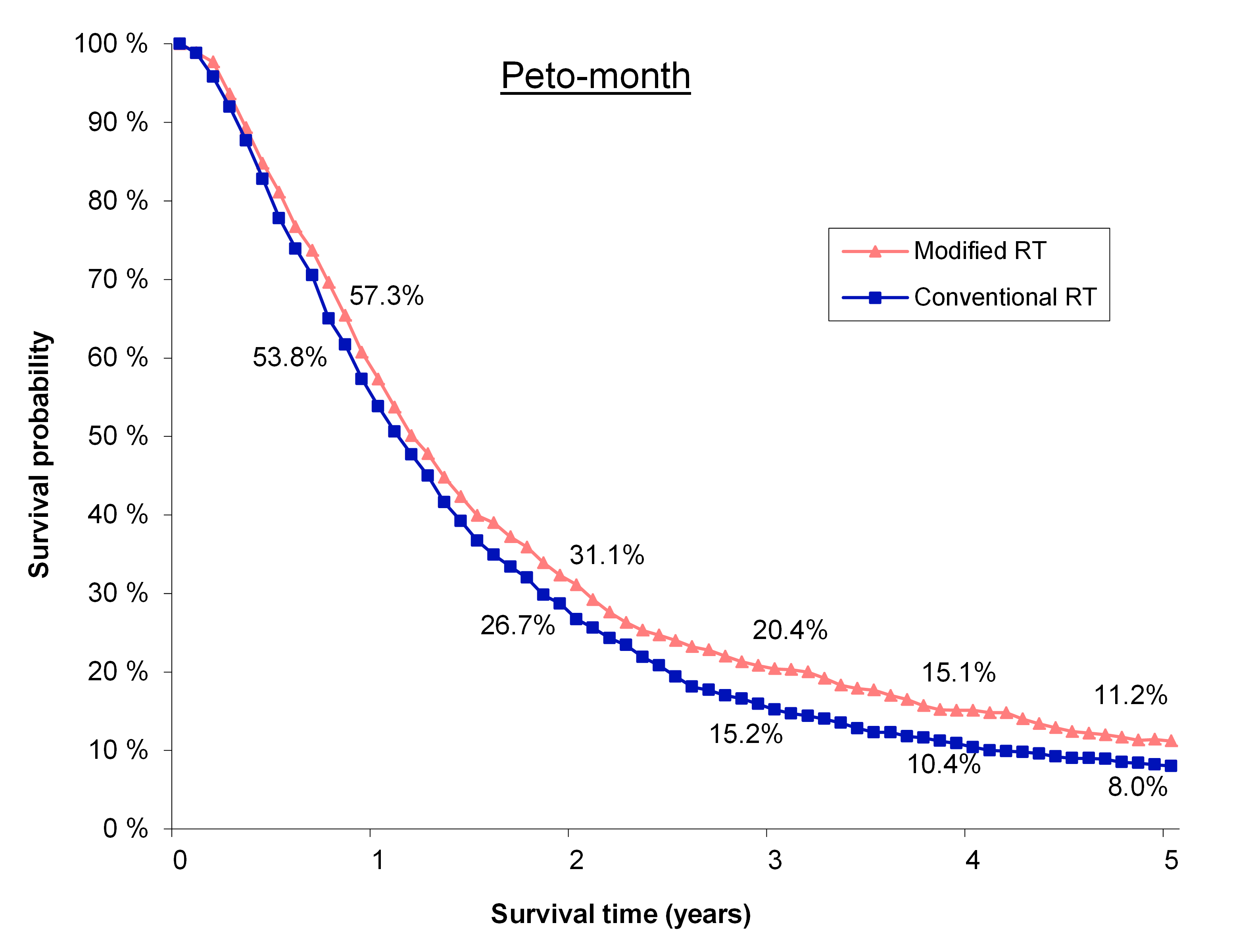

Supplement: S2 Fig — (TIF) [file pone.0150032.s002.tif]

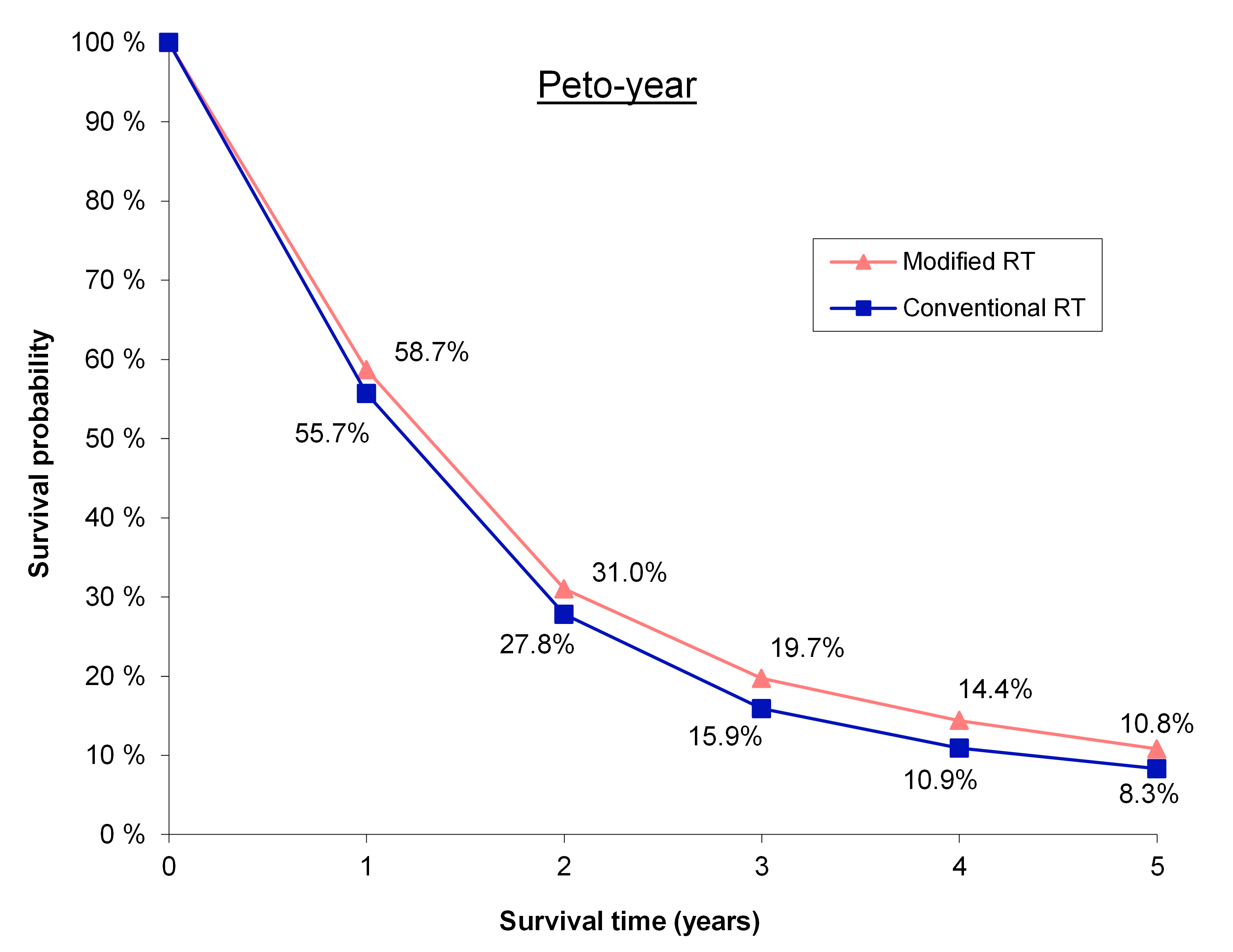

Supplement: S3 Fig — (TIF) [file pone.0150032.s003.tif]

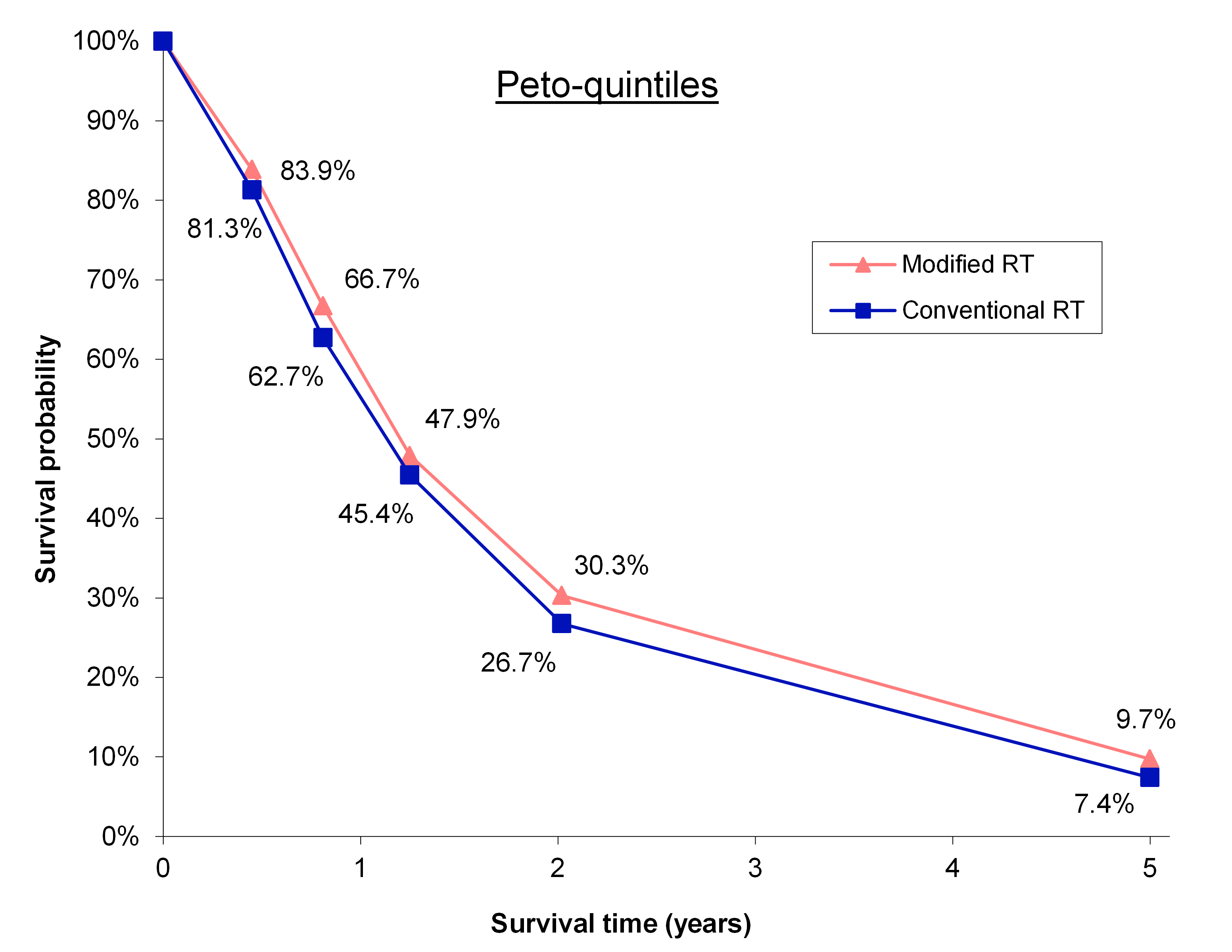

Supplement: S4 Fig — Estimations were done every 355 deaths: at 0.45 year, 0.81 year, 1.25 year, 2.02 years, 5 years and an extra point estimation for patients who died after 5 years. (TIF) [file pone.0150032.s004.tif]
