# Supplementary material for: Difference in Restricted Mean Survival Time for Cost-Effectiveness Analysis Using Individual Patient Data Meta-Analysis: Evidence from a Case Study
Source: PLoS One. 2016 Mar 9;11(3):e0150032. doi: 10.1371/journal.pone.0150032 (PMC4784740; doi:10.1371/journal.pone.0150032)
Supplement: S1 Supporting Information — (PDF) [file pone.0150032.s005.pdf]

S1 Supporting Information

Methodological details

Approach 1: Area between the two pooled survival curves

- **Stewart-Parmar:**

Stewart and Parmar methodology allows one to estimate the pooled survival curve for the experimental arm $S_{Exp,pooled}(t)$ using the pooled hazard ratio (inverse variance weighted average) and the naive Kaplan-Meier survival curve in the control group $S_{Control,KM}(t)$. The pooled survival curve for the control arm $S_{Control,pooled}(t)$ is simply set equal to $S_{Control,KM}(t)$:

$$S_{Control,pooled}(t) = S_{Control,KM}(t) \qquad \text{(S1)}$$

$$S_{Exp,pooled}(t) = S_{Control,KM}(t)^{HR_{pooled}} \qquad \text{(S2)}$$

- **Peto:**

In the Peto method, survival probabilities are estimated at predetermined time intervals i (actuarial method) based on survival probability p_i of the whole population and a pooled hazard ratio $HR_{pooled,i}$ which can vary between time periods.

$$p_i = \exp(-\frac{D_i}{PP_i}) \qquad \text{(S3)}$$

Where D_i is the number of deaths during period i and PP_i the total number of person-periods at period i. One person-period is equivalent to one person-year when the time interval chosen is one year.

$$\ln(HR_{pooled,i}) = \frac{\sum_{j=1}^{N} \frac{\ln(HR_{i,j})}{Var\left[\ln(HR_{i,j})\right]}}{\sum_{j=1}^{N} \frac{1}{Var\left[\ln(HR_{i,j})\right]}} \qquad \text{(S4)}$$

Where $HR_{i,j}$ are the hazard ratios estimated at time i in trial j through the use of the log-rank observed minus expected number of deaths and its variance.

Survival probabilities at each period i in the control arm ($p_{Control,i}$) and in the experimental arm ($p_{Exp,i}$) are estimated as follow:

$$p_{Control,i} = p_i - \left[0,5 \times p_i \times (p_i - 1) \times \ln\left(HR_{pooled,i}\right)\right] \qquad \text{(S5)}$$

$$p_{Exp} = p_i + \left[0,5 \times p_i \times (p_i - 1) \times \ln\left(HR_{pooled,i}\right)\right] \qquad \text{(S6)}$$

The survival probability at time t in each arm is the product of the probabilities across periods: $$S_{Control,pooled}(t) = \prod_{i=1}^{n_i} p_{Control,i} \quad \text{and} \quad S_{Exp,pooled}(t) = \prod_{i=1}^{n_i} p_{Exp,i} \qquad \text{(S7)}$$

Approach 2: Pooling differences in restricted mean survival time

The method to estimate the overall difference in restricted mean survival time (rmstD) aggregating the $rmstD_j$ estimated in each of the N trials is developed below with a fixed-effect model:

$$rmstD = \frac{\sum_{j=1}^{N} \frac{rmstD_j}{Var(rmstD_j)}}{\sum_{j=1}^{N} \frac{1}{Var(rmstD_j)}} \quad \text{and} \quad Var[rmstD] = \left[\sum_{j=1}^{N} \frac{1}{Var[rmstD_j]}\right]^{-1} \qquad \text{(S8)}$$

With difference in restricted mean survival time in each trial:

$$rmstD_j = \int_0^{t^*} S_{Exp,j}(t).dt - \int_0^{t^*} S_{Control,j}(t).dt = \hat{\mu}_{Exp,t^*,j} - \hat{\mu}_{Control,t^*,j} \qquad \text{(S9)}$$

$\hat{\mu}_{Exp,t^*,j}$ and $\hat{\mu}_{Control,t^*,j}$ are the restricted mean survival times estimated in trial j, respectively for the experimental (Exp) and for the control arm.

$Var[rmstD_j]$ in each trial j can be estimated based on the following formula (Karrison T. *Control Clin Trials* 1997;18:151-67):

$$rmstD_j = \hat{\mu}_{Exp,t^*,j} - \hat{\mu}_{Control,t^*,j} \;\Rightarrow\; Var\lfloor rmstD_j \rfloor = Var\lfloor \hat{\mu}_{Exp,t^*,j} \rfloor + Var\lfloor \hat{\mu}_{Control,t^*,j} \rfloor \qquad \text{(S10)}$$

Where the variances of mean survival time are estimated (Klein JP, Moeschberger ML. 1997. Survival Analysis: techniques for censored and truncated data, 1st ed. New York: Springer-Verlag):

$$Var\lfloor \hat{\mu}_{arm,T,j} \rfloor = \sum_{i=1}^{D_j} \left[\int_{t_{i,j}}^{t^*} S_{arm,j}(t).dt\right]^2 \frac{d_{i,j}}{Y_{i,j}(Y_{i,j} - d_{i,j})} \quad \text{where arm = Exp or Control} \qquad \text{(S11)}$$

In each trial j: D_j is the number of distinct event times ($t_{1,j} < t_{2,j} < \ldots < t_{D,j}$), $Y_{i,j}$ is the number of individuals who are at risk at time $t_{i,j}$ and $d_{i,j}$ is the number of events at time $t_{i,j}$.

To calculate the overall rmstD (equation (S8)), $S_{arm,j}(t)$ has then to be replaced by its estimator depending on the survival analysis method chosen (Kaplan-Meier and exponential in our empirical application) in the equations (S9) and (S11)

- **Pooled Kaplan-Meier:**

Replacing $S_{arm,j}(t)$ by the Kaplan-Meier estimator $\hat{S}_{arm,j}(t)$, it leads to:

$$rmstD_j = \hat{\mu}_{Exp,t^*,j} - \hat{\mu}_{Control,t^*,j} = \sum_{i=1}^{D_j} \hat{S}_{Exp,j}(t_{i,j}) \times (t_{i+1,j} - t_{i,j}) - \sum_{i=1}^{D_j} \hat{S}_{Control,j}(t_{i,j}) \times (t_{i+1,j} - t_{i,j}) \quad \text{(S9-bis)}$$

$$Var\left[\hat{\mu}_{arm,T,j}\right] = \frac{m_j}{m_j - 1} \sum_{i=1}^{D_j - 1} \frac{a_{i,j}{}^2}{Y_{i,j}(Y_{i,j} - d_{i,j})} \quad \text{(S11-bis)}$$

where $a_{i,j} = \sum_{l=i}^{D_j - 1} \hat{S}_{arm,j}(t_{l,j}) \times (t_{l+1,j} - t_{l,j})$ and $m_j = \sum_{l=1}^{D} d_{l,j}$

- **Pooled Exponential**

Replacing $S_{arm,j}(t)$ by the exponential estimator $\hat{S}_{arm,j}(t) = e^{-\lambda_{arm,j}.t}$, it leads to:

$$rmstD_j = \int_0^{t^*} e^{-\lambda_{Exp,j}.t}.\mathrm{dt} - \int_0^{t^*} e^{-\lambda_{Control,j}.t}.\mathrm{dt} = \frac{1 - e^{-\lambda_{Exp,j}.t^*}}{\lambda_{Exp,j}} - \frac{1 - e^{-\lambda_{Control,j}.t^*}}{\lambda_{Control,j}} \quad \text{(S9-ter)}$$

and $$Var\left[\hat{\mu}_{arm,T,j}\right] = \sum_{i=1}^{D} \left[\frac{e^{-\lambda_{arm,j}.t_i} - e^{-\lambda_{arm,j}.t^*}}{\lambda_{arm,j}}\right]^2 \frac{d_{i,j}}{Y_{i,j}(Y_{i,j} - d_{i,j})} \quad \text{(S11-ter)}$$
