# Supplementary material for: Difference in Restricted Mean Survival Time for Cost-Effectiveness Analysis Using Individual Patient Data Meta-Analysis: Evidence from a Case Study
Source: PLoS One. 2016 Mar 9;11(3):e0150032. doi: 10.1371/journal.pone.0150032 (PMC4784740; doi:10.1371/journal.pone.0150032)
Supplement: S1 Table — CHART: Continuous Hyperfractionated Accelerated Radiation Therapy; CHARTWEL: CHART Week-End Less; CI: confidence interval; CT: chemotherapy; ECOG: Eastern Cooperative Oncology Group; HR: Hazard ratio for Modified RT versus Conventional RT; MAR-LC: Meta-Analysis of Radiotherapy in Lung Cancer; NCCTG: North Central Cancer Treatment Group; PCMI: Peter MacCallum Institute; RTOG: Radiation Therapy Oncology Group; RT: Radiotherapy; *: see reference [19] for further details and for the trials references. (PDF) [file pone.0150032.s006.pdf]

Trials*	Number of patients randomized	HR [95% CI]
PMCI 88C091	101	0.97 [0.65-1.44]
PMCI 88C091 CT	107	1.26 [0.86-1.86]
CHART	563	0.78 [0.66-0.94]
ECOG 2597	119	0.75 [0.51-1.10]
CHARTWEL	300	1.00 [0.79-1.28]
CHARTWEL CT	106	0.74 [0.48-1.13]
Gliwice 2001	58	0.90 [0.52-1.54]
NCCTG 902451	74	0.64 [0.39-1.05]
NCCTG 942452	246	0.95 [0.73-1.24]
RTOG 8808	326	0.92 [0.74-1.15]
**Overall**	**2000**	**0.88 [0.80-0.97]**

S1 Table. List of trials included in the Meta-Analysis of Radiotherapy in Lung Cancer

CHART: Continuous Hyperfractionated Accelerated Radiation Therapy; CHARTWEL: CHART Week-End Less; CI: confidence interval; CT: chemotherapy; ECOG: Eastern Cooperative Oncology Group; HR: Hazard ratio for Modified RT versus Conventional RT; MAR-LC: Meta-Analysis of Radiotherapy in Lung Cancer; NCCTG: North Central Cancer Treatment Group; PCMI: Peter MacCallum Institute; RTOG: Radiation Therapy Oncology Group; RT: Radiotherapy; *: see reference [19] for further details and for the trials references
